# Health system challenges to hypertension and related non-communicable diseases prevention and treatment: perspectives from Ghanaian stakeholders

**DOI:** 10.1186/s12913-019-4571-6

**Published:** 2019-10-15

**Authors:** Amos K. Laar, Alma J. Adler, Agnes M. Kotoh, Helena Legido-Quigley, Isabelle L. Lange, Pablo Perel, Peter Lamptey

**Affiliations:** 1 Department of Population, Family, & Reproductive Health, School of Public Health, University of Ghana, LG 13, Legon, Accra, Ghana; 2 Department of Non-communicable Disease Epidemiology, London School of Hygiene & Tropical Medicine, Keppel St, London, WC1E 7HT UK; 3 Department of Global Health and Social Medicine, Harvard Medical School, Boston, MA USA; 4 Department of Infectious Disease Epidemiology, London School of Hygiene and Tropical Medicine, Keppel Street, London, WC1E 7HT UK; 5 Family Health International 360, DC, Washington, WA USA; 6 Saw Swee Hock School of Public Health, National University of Singapore, 12 Science Drive 2, #10-01, Pulau Ujong, Singapore

**Keywords:** Hypertension prevention and treatment, Non-communicable diseases, Stakeholders, Challenges, Ghana

## Abstract

**Background:**

Hypertension, itself a cardiovascular condition, is a significant risk factor for other cardiovascular diseases. Hypertension is recognized as a major public health challenge in Ghana. Beginning in 2014, a collaborative team launched the community-based hypertension improvement program (ComHIP) in one health district in Ghana. The ComHIP project, a public-private partnership, tests a community-based model that engages the private sector and utilizes information and communication technology (ICT) to control hypertension. This paper, focuses on the various challenges associated with managing hypertension in Ghana, as reported by ComHIP stakeholders.

**Methods:**

A total of 55 informants – comprising patients, health care professionals, licensed chemical sellers (LCS), national and sub-national policymakers – were purposively selected for interview and focus group discussions (FGDs). Interviews were audio-recorded and transcribed verbatim. Where applicable, transcriptions were translated directly from local language to English. The data were then analysed using two-step thematic analysis. The protocol was approved by the two ethics review committees based in Ghana and the third, based in the United Kingdom. All participants were interviewed after giving informed consent.

**Results:**

Our data have implications for the on-going implementation of ComHIP, especially the importance of policy maker buy-in, and the benefits, as well as drawbacks, of the program to different stakeholders. While our data show that the ComHIP initiative is acceptable to patients and healthcare providers – increasing providers’ knowledge on hypertension and patients’ awareness of same- there were implementation challenges identified by both patients and providers. Policy level challenges relate to task-sharing bottlenecks, which precluded nurses from prescribing or dispensing antihypertensives, and LCS from stocking same. Medication adherence and the phenomenon of medical pluralism in Ghana were identified challenges. The perspectives from the national level stakeholders enable elucidation of whole of health system challenges to ComHIP and similarly designed programmes.

**Conclusions:**

This paper sheds important light on the patient/individual, and system level challenges to hypertension and related non-communicable disease prevention and treatment in Ghana. The data show that although the ComHIP initiative is acceptable to patients and healthcare providers, policy level task-sharing bottlenecks preclude optimal implementation of ComHIP.

## Background

Raised blood pressure, has since 1972 been acknowledged as a risk factor for cardiovascular disease and mortality [1]. It is one of the top ten causes of death globally; estimated in 2010 to have contributed to 9.4 million deaths [2]. Data from the 2005, 2010, and 2015 Global Burden of Disease (GBD) studies call on stakeholders to address the heavy burden that raised blood pressure exacts on global public health [3–5]. The 2015 GBD analysis identified raised blood pressure (commonly defined as systolic blood pressure over 115), among four others (smoking, high blood sugar, high body mass index, and childhood undernutrition), to be the world’s leading risk factors for premature death [5]. If not managed, hypertension (commonly defined as a systolic blood pressure ≥ 140 or diastolic blood pressure ≥ 90), can cause several health problems including heart attack, stroke, heart or kidney failure, and blindness [6]. Raised blood pressure affects approximately 22% of adults over the age of 18 years, with low and middle-income countries (LMIC) bearing the largest burden where it is estimated that by 2020, 80% of the cardiovascular disease burden will reside [6]. The World Health Organization (WHO) further notes that by 2020, three out of four of all deaths in Africa may be attributable to raised blood pressure [6].

Despite the high global burden of hypertension, awareness and treatment are low, with only 34% of Africans aware of their hypertension, 31.3% receiving treatment and only 6.5% with their hypertension considered to be under control [7]. In Ghana, between one-quarter to one-third of adults have hypertension, though only a minority of those with the disease are aware of their status: approximately 37% of women and 20% of men according to recent local surveys and reviews [8–13]. These studies show that among those with hypertension in Ghana, even a smaller proportion, between 1.7 and 12.7%, have their hypertension controlled. Unfortunately, having traditionally focused its resources on addressing communicable diseases and maternal and child health, there is inertia in refocusing and reprioritization of the Ghana healthcare system to emerging challenges.

The current paper presents perspectives of Ghanaian stakeholders involved in a multi-facetted community-based hypertension improvement initiative – herein referred to as the community-based hypertension improvement program (ComHIP). The paper reports a nuanced understanding of the patient-level and contextual challenges associated with managing hypertension and related NCDs in a Ghanaian district.

### Ghana’s health service delivery system challenges and priorities

To provide a nuanced understanding of the various challenges associated with managing NCDs in Ghana, an introduction to the challenges and priorities of Ghana’s health service delivery system is necessary. Since the founding of the current national health system – post independence, key actors in the health policy making process have tended to be the same actors that play key roles in health agenda setting, design, adoption, implementation and sustainability in Ghana. The main governmental actors have been the Ministry of Health (MOH) and key agencies such as the Ghana Health Service (GHS). Lately, however, other actors such as multilateral agencies, bilateral/development partners, and local faith-based health service delivery actors, such as the Christian Health Association of Ghana (CHAG) have emerged. The local civil society, and a few lay advocates, although wielding minimal influence, advocate for financial resources to be made available for priority interventions. Despite some successes in terms of such advocacy, a number of challenges continue to confront Ghanaian health policy formulation and implementation – especially prioritization and resource allocation. Although the sector considers NCDs an emerging public health problem, longstanding health challenges such as infectious diseases, maternal, neonatal and new-born deaths, malaria, and sanitation continue to receive a significant proportion of health system allocation.

Aside from the prioritization and system-level challenges, there are acknowledged inequities in health service delivery or receipt of same. To address some of these inequities, the National Health Insurance Scheme (NHIS), Ghana’s first social protection efforts that addressed NCDs, was born [14]. The NHIS aims to increase access to health care and improve the quality of basic health care services for all persons living in Ghana, but especially for the poor and the vulnerable. Prior to the scheme, the majority of health care costs were paid out-of-pocket by individuals and families (a system that was referred to as ‘cash-and-carry’). Currently both public and private health care providers throughout Ghana provide healthcare through the scheme [15]. The scheme’s benefit package covers about 95% of diseases in Ghana including hypertension.

### The ComHIP project

With funding from the Novartis Foundation, Basel, Switzerland, FHI360 in 2014 collaborated with the Ghana Health Service (GHS) to launch the community-based hypertension improvement program (ComHIP) in one health district in Ghana (the Lower Manya Krobo Municipality, Eastern Region). ComHIP, which delivers a series of implementations, is being evaluated for impact and cost-effectiveness. The intervention comprises a package of evidence-based interventions that were adopted, adapted, and implemented in Ghana. Key components of the intervention include community-based blood pressure screening, management of hypertensive clients by community-based nurses (community cardiovascular disease nurses), blood pressure monitoring and supportive messaging to clients and service providers using information and communication technology (ICT) tools. Envisaged to be implemented by community pharmacists, and Licensed Chemical Sellers (LCS), dispensing of anti-hypertension drugs could not be implemented. The overall community-based intervention and evaluation strategies are detailed in [12]. Currently referred to as over-the-counter medicine practitioners, LCS in Ghana are permitted to supply retail restricted drugs other than Class A or B drugs. Community pharmacists, per local guidelines are higher cadre private service providers. They supply medicines in accordance with a prescription or, when legally permitted, sell them without a prescription. Unlike the LCS, the main activities of community pharmacists include processing of prescriptions, care of patients, monitoring of drug utilization, and health promotion.

Prior to ComHIP, the FHI360, the GHS, and others developed and implemented a general education and CVD screening programme in the district in which ComHIP was implemented. As part of this earlier programme, counselling, health education and lifestyle modification interventions were provided alongside screening for hypertension. The study revealed hypertension as a major problem in the setting – reporting a prevalence of 32% among females and a little over 34% among males [16]. Baseline data from the ComHIP project show similar prevalence of hypertension- 32.4%. The data further show that although 46.2% of those diagnosed to be hypertensive were aware of their hypertension status, only 9% were treated and 1.3% of patients had their blood pressure under control. Furthermore, we found in-depth knowledge of hypertension risk factors to be low despite the high level of knowledge about the burden of the condition. Overall, close to 50% of the adult population were overweight or obese [12]. Thus, challenges revealed by these surveys is the high mismatch between hypertension knowledge and hypertension diagnosis, treatment or control. The existence of low blood pressure control rates in the intervention district despite previous generalized education programs [16] suggests that such programs have little impact on hypertension control.

Following the baseline study, we qualitatively assessed ComHIP implementation barriers, enablers, and factors influencing adherence (this is reported elsewhere; see Adler et al., forthcoming). This paper focuses on health system factors, service provision factors and service user factors that impact hypertension prevention, management, and control in Ghana. Publicizing the findings not only shares implementation experiences, it also offers stakeholders the opportunity to appreciate identified challenges and propose solutions as they explore scale up options. Beyond ComHIP, the study findings may provide guidance to similarly-designed interventions.

## Methods

### Study design

The ComHIP evaluation is conducted in two peri-urban districts in Ghana – the Lower Manya Krobo Municipality (intervention site), and the Akuapim South District (comparison site). The comparison district, which provides the current GHS hypertension services, is distant enough to minimize contamination. The evaluation employs before-after population-based surveys (in intervention and control districts), and a prospective cohort study in the intervention district (for details, see Lamptey et al. [12]. Conducted among hypertensive patients enrolled in the cohort are a series of patient centeredness surveys and qualitative investigations among various ComHIP stakeholders.

The design of the qualitative study is informed by the WHO Health Systems Assessment Framework [17] and the WHO’s identification of five interacting dimensions that affect adherence [18]. This framework depicts health systems using six interrelated units or building blocks. These are service delivery, health workforce, information, medical products, vaccines and technologies, financing, and leadership/governance. Having been previously used to analyse the relationships between health system levels for cardiovascular or hypertension conditions [19], this framework facilitated the identification of systems-level barriers to hypertension care in Ghana.

The five interacting dimensions of adherence [18] encompass social and economic factors, health systems factors, condition-related factors (in the current case, hypertension or NCD), treatment-related factors, and patient-related factors. As hypertension care and associated challenges are determined by the interplay of several factors, the study tools were fashioned with these dimensions in mind. The two frameworks facilitated the exploration and appreciation of the multiplicity of factors impacting hypertension care. Thus system-level factors such as governance and prioritization of NCDs within the Ghana health service delivery system; human resource constraints, policy-informed task-sharing challenges; and patient-level challenges such as life-long management of hypertension, adherence to medications and medication side effects; and socio-political questions of medical pluralism were investigated.

### Study respondents

A total of 55 informants were purposively selected to represent the characteristics of each category of study participants (see Table 1 below). We selected registered patients and ensured a mix of gender, age, district, and socio-economic status. Health care providers were selected to ensure representativeness of hospitals, and LCS to ensure their representativeness of the studied communities. Policymakers from the Ministry of Health/Ghana Health Service were selected based on the relevance of their position within NCD control. They included the National Programme Managers, Regional Directors of Health Services, Policy, Planning, Monitoring and Evaluation Officers.

**Table 1:** Categories of informants

Informants	Semi-structured interviews	Focus Group Discussion (FGD)	Total
Patients	15	16	31
Health care professionals	10	–	10
Licensed chemical sellers	–	7	7
Policymakers	7	–	7
Total	32	23	55

### Study tools development, field staff recruitment, training, and data collection

As described above, the design and the tools of this qualitative study benefit from the WHO Health Systems Assessment Framework [17], and the WHO resource relating to five interacting dimensions of medication adherence [18]. The framework and associated questions captured in our tool facilitated our identification of health systems-level barriers to hypertension care in Ghana. We developed five separate, yet related tools – one tool for healthcare professionals (semi-structured interview guide); this tool covered topics such as: a) experience and tasks; b) how treatment and care is provided and coordinated; c) how treatment has changed with the programme; d) their relationships with patients; and e) areas for improvement at the health systems level). The second tool was used to engage health policymakers (a semi-structured interview guide). The interviews with policy-makers were tailored to their expertise and position. The interviews focused on understanding NCD programmes, the key barriers in the implementation of hypertension programmes, key health systems-level facilitators, who the key actors are, aspects that need improvement, and the commitment to scaling up the ComHIP programme. For hypertensive patients, we developed both a semi-structured interview guide, and a focus group discussion (FGD) guide. The specific interview and focus group discussion topics for hypertensive patients enrolled in the ComHIP intervention include: a) how treatment and prevention of hypertension is provided; b) health care experience and recommendations; c) knowledge of hypertension and its diagnosis; d) health system barriers and facilitators to accessing services and receiving care for hypertension; e) factors influencing adherence. For licensed chemical sellers (focus group discussion guide), the topics included a) their experience and perceptions of the programme b) scalability. These tools are consolidated and appended to the manuscript as a supplementary material (see Additional file 1).

Using the above tools, three experienced multilingual qualitative field researchers –fluent in English, Krobo or Twi carried out face-to-face semi-structured interviews and FGDs. Two researchers (co-authors AJA and AK) conducted an intensive one-week training session for the qualitative field staff to ensure that interviewers understood the objectives of the study and the questions. They were trained to respond to any sensitive situations or signs of distress with appropriate wording, supportive statements and avoidance of excessive probing. The training included extensive role plays, and trialing of the tools. The issues and experiences from the trial process were incorporated into the tool prior to actual data collection.

Based on participant preference, interviews and FGDs were conducted in English, Krobo or Twi and audio recorded. The categories of the study participants are summarized in Table 1. We interviewed 15 hypertensive patients enrolled in the intervention, 10 health care professionals (seven CVD nurses, one pharmacist, one physician assistant, and one physician), seven health policymakers. We conducted two FGDs with a total of 16 patients (one group of men, and one of women). We conducted a third FGD with seven LCS.

### Data analysis and quality assurance

The interviews were audio recorded and transcribed verbatim. The transcriptions were translated directly from the local languages to English by experts in English and Krobo or Twi, and wherever possible by the individual who conducted the interview. Our quality assurance measures included recruitment of experienced multilingual qualitative researchers, and conducting a one-week training on the study methods, tools, and ethics. During the transcription process, co-author AK randomly sampled 10% of the translated transcripts and compared them with the original recordings. The data were then analysed using thematic analysis techniques employing two steps. We started with a-priori chosen themes based on the ComHIP intervention themes. We coded transcripts based on both the a-priori analysis and emergent and divergent themes that came from the analysis. We then looked to find overarching concepts in which the themes were interconnected. Co-authors AJA, AK, AKL, and field assistants met to discuss major themes and issues that were not clear in the transcriptions.

### Ethics

Our study protocol received ethics approval from the Institutional Review Boards (IRBs) of the London School of Hygiene and Tropical Medicine (LSHTM) - Observational / Interventions Research Ethics Committee (Ref: 10152), the Ghana Health Service – Ghana Health Service Ethics Review Committee (Ref: GHS-ERC 04/01/15) and the Noguchi Memorial Institute for Medical Research, University of Ghana (Ethics clearance # IRB00001276). Participating in the study was preceded by a written informed consent processes, which communicated to prospective participants that their participation in the study was voluntary. It also outlined measures that the researchers would put in place to ensure privacy, anonymity and confidentiality. Briefly, the measures instituted by the study team to ensure that participants’ confidentiality is maintained included the following. Personal identifiers were not included in study reports and manuscripts. All study records were secured to the extent provided by local and international regulations. Data collection forms were identified by codes; all records containing names or other personal identifiers, such as informed consent forms were stored separately. The required anonymization procedures were implemented prior to depositing transcripts in Harvard Dataverse (an open access repository). Regarding privacy, interviews were conducted in private and comfortable spaces that the participant deemed acceptable. All participants consented to the publication of their data.

## Results

Presented below are both health system level challenges and patient level associated with managing hypertension in a district benefiting from the ComHIP intervention. The health system level challenges span governance and prioritization of NCDs within the Ghana Health Service delivery system; human resource and task-sharing challenges; and challenges of medical pluralism of illness and treatment. Hypertension-specific challenges relate to integrating hypertension prevention and screening within the health system, and provision of hypertension prevention and treatment at primary care facilities. Also presented are several patient-level challenges including inadequate understandings of medications, medication adherence, and side effects.

### Governance and prioritization of NCDs within the Ghana health service delivery system

Key stakeholders of the GHS (national, regional, and district level policymakers, health professionals and clients) shared their perspectives on the systemic health system-wide challenges as a whole, and in particular, management of NCDs within the Ghana health delivery system. In particular, the perspectives from the national level policymakers enabled elucidation of prevailing system-wide challenges (beyond ComHIP intervention district) such as a mismatch between prioritization of NCDs and current level of funding for NCDs interventions. Policymakers consider NCDs as an emerging public health problem. Longstanding health challenges such as infectious diseases, maternal, neonatal and new-born deaths, malaria, and sanitation continue to be health sector priorities. The current level recognition of NCDs is reflected in the government’s financing strategies. A significant proportion of health system allocation goes to maternal, child health and infectious disease programmes. However, the pendulum, in a foreseeable future, is likely to swing toward NCDs, as their burden, particularly hypertension-related morbidity and mortality are increasing. A GHS official at the district level explained the situation as follows:

*Ghana being a developing country, our focus is more on how to combat communicable diseases. Now the number of NCDs is also increasing and the large number of clients that come to the facilities is a matter of concern. Previously, we see older people having the disease. But now we find younger people coming. Also, many people come with stroke or die suddenly. The increasing burden of hypertension in our communities and facilities is gradually changing health sector prioritization. (*District-level GHS official).

Beginning in 2012, the Ministry of Health developed a national policy for the Prevention and Control of NCDs in Ghana [20]. An accompanying strategy, the strategy for the Management, Prevention and Control of Chronic Non-Communicable Diseases in Ghana 2012–2016 [21] was also developed. The NCD strategy outlines, amongst others, health promotion, community engagement and outreach and covers advocacy, creating awareness, screening for early detection, diagnosis and management and research.

Although Ghana’s national policy and accompanying strategy for NCDs have been in existence since 2012, inadequate logistics and financial resources limit their implementation. Policymakers revealed that unlike infectious diseases, or maternal and child health issues, there are no defined sources of funds from the Ghanaian government for awareness creation, screening and prevention of hypertension. Policymakers reported that the major challenge to the control of hypertension in Ghana is the lack of awareness, or knowledge (on the part of the populace) as well as the GHS’ lack of resources. Most of the GHS interviewees noted that GHS lacked funds for awareness creation and community-based education. Health promotion is generally carried out through media campaigns, primarily radio, where knowledgeable persons discuss risk factors for specific NCDs, clinical presentation, treatment, and possible complications. However, these programmes do not provide the opportunity for people to call in and ask questions and clarify issues. Many policymakers noted that at public health facilities, only opportunistic screening is done due to a lack of screening facilities’ equipment and staff. This is seen through the strain that the current ComHIP programme has put on the hospitals in the Lower Manya Krobo (the ComHIP intervention district). There is a concern that increased screening may increase demand to greater than the health services’ capacity to treat the screened individuals.

### Human resource constraints and task-sharing challenges

Related to inadequate logistics and financial resources is the problem of human resource. Ghana’s precarious health workforce situation, particularly its inadequate number of doctors, is detrimental to effective health delivery including NCD management. Three policymakers independently noted that the NCD unit, just as other sectors of GHS, has serious human resource challenges. Throughout the country, particularly in remote settings, limited number of trained personnel exacerbate already existing service delivery inequities. A substantial challenge faced by the ComHIP project was the shortage of trained staff (particularly doctors) with hypertension medication prescribing authority and expertise for hypertension treatment and management. A CVD nurse’s complaints and the views of a service user participating in a focus group illustrate this challenge.

*Unlike the situation with other drugs such as anti-malaria drugs and some antibiotics in pregnancy we prescribe, only doctors give prescription in the case of NCDs. This leads to long waiting and difficulties accessing needed medications. (*Community based CVD nurse)

This complaint (below) about wait times at hospital during visits for ordinary monitoring of blood pressure is emblematic of others recorded during the focus groups. Discussants of the focus groups offer task-sharing suggestions to address this problem.

*For me, my major challenge is that sometimes you are just in need of your BP drugs but you will get to the hospital very early and leave there very late, so to me if they can arrange and group BP patients at one side so that you join that queue and go for your medicine immediately. Perhaps the nurses who counsel us can be permitted to have the medicines so that once in a month when we go to see them they can give us the medicine. That will be simple. Imagine going to join the long queue just for medicine; I prefer to go to the drug store to buy them myself.* (A female FGD participant*)*

The human resource challenge impacts nationwide integration of hypertension prevention and screening within the health system. Aside from a shortage of experts, there are challenges of their distribution across the country with varied regional and district disparities. A common theme that emerged from conversations was that facilities in deprived areas are severely understaffed. Many doctors are not willing to accept postings to rural areas where more health care professionals are needed. While the number of medical schools is increasing as part of the national strategy to train more staff locally, there are huge inequities of trained personnel, particularly doctors with expertise. Many of them move to more desirable areas or leave the GHS.

*Deprived, hard-to-reach areas (sometimes referred to, in Ghanaian parlance, as “overseas”) are severely understaffed. When serious cases show up at the district hospitals, they are referred to regional hospitals. Currently, these clinics are much less common, so if one does not live near a district or regional hospital, one is unlikely to get specialist care for NCDs including hypertension and other chronic conditions. That there is unequal geographical distribution of healthcare professionals is no news to us.* (National-level policy maker)

This inequitable distribution of high cadre health workforce precludes integration of hypertension prevention and care into lower level service delivery points where providers do not have antihypertensive prescribing authority or lack hypertension management experience. Alluded to earlier, unlike other conditions, in Ghana management of hypertension is hospital-based with doctors as prescribers, and service provided by specialised clinics run once or twice a week at district and regional hospitals. Lower cadre service providers such as nurses do not have prescribing or dispensing power.

### Hypertension treatment and access-related challenges

Aside from task-sharing access-related challenges described above, financial access is an important challenge. Of note, hypertension is covered by the NHIS, therefore, all patients theoretically have access to hypertension care in Ghana. The precondition, however, is to enrol in the NHIS scheme and pay the subsidised annual premium (GH25.00 about US$6.00). Exemptions are also provided for various vulnerable groups. Despite this initiative, some health professionals in public health facilities expressed their frustration with the NHIS scheme and the challenge of accessing hypertension medications. While the assumption was that most patients had health insurance cards, many informants felt that there were still many out of pocket costs including transport. This, combined with the time spent by patients getting prescriptions, led to substantial challenges.

Others pointed to the challenges of the NHIS as having a negative impact on hypertension treatment.

In particular, the delayed reimbursement of health facilities by the National Health Insurance Authority (NHIA) seriously affects health facilities’ stock of medicines and other supplies.

*Unfortunately, health insurance has not paid our claims for close to a year now. We are in April and the last claim that was paid to all our health facilities was April last year, 2016. So that is 11 months that they haven’t paid us, and you know the drugs you procure from regional medical stores or you procure from the open market and you have to pay for the drugs and the patient knows. If he or she is on health insurance then antihypertensive drugs, the majority of them, are on the inclusion list so they are provided to him or her free of charge. Yes, when it comes to the facility level because they don’t have money to pay supplies so then they have to put in maybe a payment or co-payment. So maybe now the health insurance is not paying us so you have to pay half and the facility is also paying half just for the facility to survive, or if they don’t have drugs in some of the facilities then they will just write prescription for you to go and buy at the pharmacy shop or chemical shop. And you know some of them because it is cash when they go they may not buy it and take their drugs.* (District level nurse)

A related access challenge identified by many informants was the unavailability of essential drugs at lower level service delivery points. Community health nurses reported that some patients only took one round of medications and did not refill their prescriptions due to the cost of transport to NHIS accredited facilities. Also, many community pharmacists do not stock antihypertensive drugs, making it difficult for patients who are able to purchase them to travel longer distances to obtain them. A service provider expressed her frustration about getting medication for their patients in the following comments:

*At the community level, you may have to travel to get your medicines. What’s the point if I diagnose and I can’t give medicine? If I go to the nearest health centre and I diagnose someone to have hypertension then I’m working at the health centre, I don’t store antihypertensive drugs so I give you a prescription and you have to go to the regional hospital, that is a problem. Why doesn’t the client just go to the regional hospital for diagnosis and treatment?* (Senior member of the GHS)

### Patient-level challenges: life-long management of hypertension, adherence to medications and medication side effects

One of the biggest challenges to effective hypertension control mentioned by most informants was adherence to medications. Our results show that with the exception of a few, patients are aware of the necessity of the continuous taking of medications but do not do so. Reasons given for non-adherence included the inaccessibility and unavailability of drugs, non-acceptance of life-long management of hypertension, side effects associated with the drugs and perceptions of traditional medicines as curative.

All participants of the two focus groups knew that they should talk to their health care providers if they had any side effects or problems with any hypertension medications. No patient reported receiving incorrect doses of drugs when prompted, and no nurse knew first hand of a patient receiving the wrong dose. Both patients and nurses had concerns about side effects, which included too frequent urination and erectile dysfunction for men. Nurses cited side effects as the primary cause of non-adherence, and a few hypertensive focus group participants stated that they switched medications because of side effects.

The influence of side effects on medication adherence seemed greater in men, compared to women. One nurse noted. “*Men are more reluctant to take antihypertensive drugs because of the sexual side effects.” .* Another nurse elaborated with a clarification:

*… the main reason why males are likely to stop taking medications is side effects, while females stop if they feel their BP has gone down. They asked, ‘why should they continue taking the drugs if they are okay?’*

The above was corroborated by views of some hypertensive participants. One male participant expressed their worry about the medicine's effect on his sex life.

*I had erectile dysfunction and had told the CVD nurse, but she laughed and did not change my medicines. So, I chose to stay off the drugs for a while.*

A nurse reported that patients would wait too long to report problems with their medications:

*When they start experiencing side effects, instead of reporting immediately, they stop taking their medications and wait until their next visit*. *Some will not even come to you at all.*

We also explored the challenges associated with the life-long management of hypertension. For instance, adherence was hampered as many patients did not realize the medications were meant to be continued even after their blood pressure was lowered. One participant gave an example of stopping taking the drugs once the symptoms disappear.

*I have seen that you can’t stop the medication. I’m saying this because during Christmas I stopped taking my medication so that I could enjoy the season but after Christmas I realized that my heart started beating abnormally again. When I went to check it was around 200. The doctor told me that I have stopped taking my medication. I tried to lie but he insisted that I had stopped. He gave me some medications that I took for three days. I went back and the BP reduced to 130 and he told me it’s because I have taken the medication this time.*

A pharmacist attributed the life-long hypertension treatment challenge to polypharmacy.

*The quantity of drugs given to patients to take discourages them from adhering for a long time. The sheer number of drugs discouraged them, although it was noted that if you gave patients too few medications they would also complain.*

### Reconciling two health systems and cultures: traditional medicine vs Western medicine

The desire for curative drugs rather than management is a major barrier to adherence to antihypertensive medications. There are many traditional or herbal medications in Ghana that are perceived to cure hypertension. While antihypertensive drugs are taken for life to manage the condition, traditional/herbal medicines purport to be curative. This perception often discourages people from accessing allopathic care and if they do, continuation of the treatment for life is a challenge. According to nurses, some of their clients reported taking traditional medicines. Some of the patients also told us they took traditional medicines but did not tell the CVD nurse and admonished us not tell them. A participant said prior to ComHIP he nearly had a stroke and started taking herbal medications that were made by his brother. However, when he combined the herbal and allopathic medications, he experienced some negative effects, so he stopped taking the local one. Another participant stated that he only sought hospital care out of his concern of not having sufficient traditional medicines.

The illustrations below show the popularity of traditional medicines, which are sometimes taken concomitantly with allopathic antihypertensives or may be used as a substitute for the allopathic medications:

R: *I take the medicine but stopped because I’m taking the local one. Now I’m yet to start the foreign medication. I take it because the lady selling it told me it’s good for hypertension.*

*I: So, you combine the two?*

*R: No, the seller told me to stop the hospital one for some time so that I won’t mix the two drugs till I finish the local one. Recently when I came here, and they checked my BP they asked me if I’m taking some other form of medicine in addition to the foreign ones and I told them yes.* (Female participant)

*She used to be on the hypertensive drug and then she stopped before I met her. Some of them told me they stick to some herbal medicine because when they checked and realised the BP is okay, they stopped the hospital one. So, for me to diagnose again, it means that the herbal medicine is not working so we have to go and follow the orthodox way again.* (Community CVD nurse)

## Discussion

We explored Ghanaian stakeholders’ perceptions of a multi-facetted community-based hypertension improvement initiative with task-sharing components. This paper presents and discusses several patient-level and contextual challenges associated with the implementation of the initiative – focusing on the management of hypertension and related NCDs. The findings have implications on the implementation of ComHIP and on the design of related task-sharing strategies in Ghana. Publicizing the findings not only shares implementation experiences, it also provides stakeholders an opportunity to appreciate and address identified challenges as they explore possibilities of scaling up this initiative.

Although, our data show that the ComHIP initiative is acceptable to patients and healthcare providers (*Adler* et al*, forthcoming*) – increasing providers’ knowledge on hypertension and patients’ awareness of same, the important challenges identified by both patients and providers are worth discussing. The key policy level challenges relate to task-sharing bottlenecks. Existing policy precluded lower-cadre services providers such as nurses from prescribing or dispensing antihypertensives, and LCS from stocking them. Medication adherence, side effects, and the phenomenon of medical pluralism in Ghana are other challenges. Although focused on the ComHIP intervention currently being implemented in one health district, views from national level stakeholders are reflected in this paper. Their perspectives enable elucidation of contextual and system-wide challenges such as a mismatch between prioritization of NCDs and funding NCD interventions by the Ghana health delivery system.

### Ghana’s health service delivery system challenges and priorities

Like many LMICs, Ghana’s fragile health system is dealing with multiple burdens of disease as well as non-disease health system challenges. Thus, the emerging challenge of NCDs (whose impact on the health system is real) adds to existing layers of challenges that the country’s health system has to contend with. Ama de-Graft Aikins [22] has recently discussed the impact of CVD on primary healthcare (PHC) services in urban poor communities in Ghana – laying bare the significant unmet need for CVD care in these communities. National level stakeholders in the current study acknowledged the growing challenge of NCDs. Like other countries in the sub-region, Ghana is experiencing rapid urbanisation, accompanied by increasing levels of obesity and related NCDs [13, 23, 24]. Data from the Global Burden of Disease studies rank raised blood pressure/hypertension, high fasting plasma glucose, dietary risks, and high body mass index among the top 10 risk factors that drive the most death and disability combined. Thus, NCDs and their associated risk factors account for > 40% of total morbidity and adult mortality in Ghana. In recognition of the increasing burden of NCDs, Ghana has recently politically recognised NCDs as a pressing health concern, publishing national NCD prevention policies and strategies, which identify interventions to address them [20, 21]. Closely related to the disease burden is the challenge of funding. Although there is a growing national recognition of NCDs, particularly hypertension, there is currently no commensurate funding response. Policymakers lament that the Ghana national NCD policy and its accompanying strategy have been in place since 2012, but their implementation has been suboptimal due to lack of requisite resources and funding.

To address this funding challenge, Ghana turns to donors or development partners who fund a significant fraction of the health budget [25]. These development partners play critical roles in policy design, implementation, and funding decision-making. The policymakers we interviewed said that current funding priorities are maternal and new-born mortality, sanitation, malaria, and other infectious diseases. Thus, though NCDs are nationally recognized as a major public health challenge, they are not given priority with the necessary funds to address them. To respond to this high burden of NCDs, and yet low resources to address them, studies recommend health systems-wide strengthening, which has positive externalities on persons with NCDs, as well as their families, and communities [26–28]. Such an approach is in line with the current ComHIP model.

### Human resource and task-sharing challenges

That the Ghana healthcare delivery system is beleaguered with workforce challenges is not new and is not restricted to the domain of NCDs. Ghana currently has a one doctor to 10,450 patients ratio. This falls below the one doctor to 1320 patients WHO recommendation. All policy level informants reiterated the lack of required numbers of high calibre personnel as a major challenge. In particular, the distribution of the few qualified personnel is concentrated in a few major cities, leaving significant portions of the Ghanaian population to be attended to by low cadre health personnel. However, the problem is more serious for NCD service provision. As one moves from the few endowed urban facilities toward urban poor communities, healthcare services for NCDs, particularly CVDs, are generally not accessible, equitable, nor responsive to the needs of the vulnerable [22].

In the midst of these challenges, lie opportunities to improve NCD prevention strategies, treatment and control in Ghana. For example, effective public-private partnerships (PPP), the deployment of technology, effective exploitation of the Community-based Health Planning Services (CHPS), and task-sharing provide important frameworks to improve NCD care. To help address this gap, the MOH and GHS in recent years have endeavoured to capacitate the CHNs through the strengthening of the CHPS initiative. Currently there are about 3000 CHPS compounds in Ghana, and this is projected to increase to 6000 by 2020. CHPS were originally formed to provide education to mothers, children and households. The CHNs overseeing CHPS compounds or zones have been trained to address key areas n community health (such as infectious disease preventative care and maternal and child health services). Similar training on NCDs can enhance capacity, confidence, self-efficacy to provide basic care and support to those in need. This approach has worked in other African countries such as Cameroon and South Africa [29]. Recognizing the value of the ComHIP concept, some policymakers now see CHPS as a missed opportunity for the prevention of hypertension and NCDs . It is an attempt to bring integrated hypertension management into the community through a system of community education and hypertension screening by CHNs/CVD nurses’ referrals, and ICT support for participants. Prior to ComHIP the only possibility for accessing hypertension care in a typical Ghanaian health district was in hospitals by physicians. Given the low doctor-to-patient ratio, it is imperative that innovative methods of reorganising care – such as ComHIP – are utilised.

Notwithstanding this, the significant challenges encountered during ComHIP implementation need to be discussed. One of the challenges encountered relates to task-sharing (described as the process of enabling lay or low level healthcare personnel to fulfil a wider clinical role and used interchangeable with "task-shifting" in this paper), which has been acknowledged as a viable strategy for responding to CVDs and other NCDs in LMICs [29–32]. Ogedegbe et al. [33] in their systematic review of trials on task-shifting interventions for cardiovascular risk reduction in LMICs conclude that task-shifting strategies are appropriate and deployable in many LMICs battling with infectious and chronic diseases. The WHO, for example, recommends task sharing where access to health services is constrained by a lack of health workers [34]. Other programmes in sub-Saharan Africa have found at least limited success when nurses were trained to manage hypertension (for example in DRC [35], in Nigeria [36], in Kenya [37], and Ghana [38]. In ComHIP, whilst results on the overall programme impact and cost effectiveness are still forthcoming, the data from this and earlier qualitative analysis (Adler et al. *forthcoming*) suggest that various components of ComHIP including its task-sharing experiment was acceptable to patients and nurses involved in the programme.

However, the Ghana health policy at present does not permit nurses and other lower cadre health personnel to prescribe most medications including antihypertensives. Some of the policymakers we engaged in this study indicated the desire to expand who is able to prescribe medications. It would seem to us that this challenge is not particular to Ghana. The review by Ogedegbe et al. [33] identified barriers to task shifting in LMICs as including policy gaps on medication prescription; weak or non-existent referral systems to take care of complicated cases and the inability of non-physician providers to manage uncomplicated CVDs and their risk factors [33]. In the case of ComHIP, the inability of nurses to dispense antihypertensives was a major challenge. Perhaps, identified enablers of task-shifting such as continuous educational training and feedback from higher level health professionals; bridging hospital care to home care in order to ensure continuity of patient care; and providing explicit training tools including medication/treatment algorithms [33] need to be exploited. At least two projects in Kenya and Ghana that allowed nurses to provide antihypertensives have shown promising results [37, 38]. For instance, the Ghana study pilot-tested a task-shifting strategy for a hypertension study (TASSH), which trained community health nurses to deliver hypertension care. Although the nurses did not dispense antihypertensives because they did not have prescribing/dispensing power, they (nurses) had access to antihypertensives through the coordinating physician and could dispense them as needed based on the GHS hypertension treatment algorithm. Thus, the study was able to circumvent the legal limitations by having the coordinating physician supply the needed drugs to the nurses. While this project was successful, it does not fit into the current regulations within the GHS. In light of these findings, policies should be amended. Indeed, our study, and that by Iwelunmor et al. [39], explore stakeholders’ perspectives of task-sharing as a strategy for preventing, and managing hypertension in Ghana. Both studies show that for any task-sharing intervention to be successful, a deliberate effort must be made to not only focus on patients, or individual level characteristics, but also to consider the role systems-level variables such as policy, leadership, and stakeholder engagement play.

### Patient-level challenges including multiple understandings of illness and treatment and medical pluralism

Like many others in the sub-region, patients in Ghana have the opportunity to shop for healing from various systems/care providers: the allopathic health services delivery system, indigenous/traditional care delivery systems, as well as faith-based providers. Referred to as medical pluralism [40] or more recently, medical diversity [41], the use of multiple medical systems to address illness and wellness, has been praised and criticised at the same time [42]. The medical sociology, and anthropological literature address the subject in-depth. Baer [43] and Goldstein [44] discuss its evolving nature, persistence, resurgence and concept contestations. We do not aim to interrogate the subject further. Instead, we focus on system-level factors that motivate concommittant use of traditional remedies and modern medicine in Ghana [22, 45, 46].

As far as chronic disease care in Ghana is concerned, medical pluralism's contribution has begun to emerge [47]. Hampshire [45] interrogates medical pluralism in the context of globalization and new healing encounters in Ghana. For a long time, the practice of allopathy in Ghana is often framed to compete with alternative practices such as traditional/indigenous and faith-based healing [22]. It is common practice for community members to bypass allopathic public health facilities and access care from these alternative providers or healers. Aside from inaccessibility challenges associated with allopathic healthcare, motivations for uptake of indigenous health remedies, include trust (of indigenous healers), proximity, ease of use, previous relationships, cost-value (sometimes) and poor perceptions of the level of competence of the low-cadre allopathic health workforce [48, 49]. Awah and Phillimore [50] explore and cast important light on the tension between clinic-based demands for patients’ ‘compliance’ with treatment guidelines, including repeated strictures against resorting to ‘traditional’ medicine, and patients’ own willingness to alternate between biomedical and indigenous practitioners.

Critics of indigenous medicine point out its implications for service uptake and adherence to allopathic care. In our study, potential barriers to uptake and adherence to antihypertensives relate to the belief that traditional medicines could cure patients' hypertension. Our exploration, which involved a wider group (patients, service providers, and policymakers), concurs with the findings of de-Graft-Aikins [22], where the patients she engaged set out to seek a cure for their diabetes. Similar claims are rife in studies of other NCDs and in further African settings [47]. We note that, until all care providers in this pluralistic healthcare system are made to understand that there is as yet no cure, this phenomenon of seeking cures for ‘incurable’ health conditions will continue. Therefore, programmes such as ComHIP may learn from these explorations of local approaches to care-seeking and medication by understanding patients’ priorities, motivations and preferences when designing interventions – seeing traditional solutions as complementary to, rather than competing against, allopathic medications. The popularity of traditional remedies among ComHIP clients is in line with data from previously reported local studies [51, 52], where patients sought care from multiple outlets. For example, the Ghana Herbal Pharmacopoeia reveals that more than 70% of Ghanaians who receive allopathic care, also resort to alternative health care practices to address their health needs [53]. Another local study linked such practices to Ghana’s religious landscape and political history [52]. A relatively recent Ghanaian study reported prevalent use of non-prescription medications by persons living with HIV, a practice mostly self-initiated or implemented with the acquiescence of some healthcare providers [51]. This phenomenon has been documented in other African settings [54–57].

Like limited health workforce, limited funding, misprioritization, and restrictive prescribing policies, the phenomenon of medical pluralism is an important contextual challenge to the prevetion/treatment of hypertension and other NCDs in Ghana. The Ghana Food and Drug Authority, the Ghana Standards Authority, and other regulatory bodies need to institute robust monitoring and validation schemes that endorse or reject various healing options on the formal and informal health delivery platforms. Furthermore, efforts at improving the health literacy of the Ghanaian populace may help provide clarification on the effectiveness or otherwise of these healing options.

### Study strengths and limitations

ComHIP is one of the few comprehensive studies which deploy a multilevel combination prevention-treatment-control intervention that engages (patients, community, and healthcare personnel), educates (patients and family), and uses supportive tools to increase hypertension literacy, service access, service uptake, and linkages to care.

This qualitative study involved all the actors and components of ComHIP. In doing so, it has been possible to identify enablers and bottlenecks to implementation and future scale up. The findings, however, need to be read along with the following limitations. First, the data presented in this paper derives from purposively sampled ComHIP stakeholders and thus precludes generalizability. It ought to be noted however, that, typical of qualitative inquiries, the study was not intended produce evidence generalizable to other settings. Second, although the qualitative research assistants were trained to handle courtesy bias or socially desirable responses on the part of all respondents, we are not able to wholly rule them out. Despite these limitations, this paper sheds light on important challenges to hypertension and other NCD prevention, treatment, and control in Ghana. These have the potential to inform research uptake, as well as provide guidance to similarly-designed interventions.

## Conclusions

Taken together, this paper reveals two layers of challenges associated with hypertension and related NCDs prevention and treatment in Ghana. These are the healthcare system-level challenges relating to governance and prioritization of NCDs within the Ghana Health Service care delivery system; human resource constraints, task-sharing bottlenecks; and patient-level challenges relating to non-adherence to medications and medication side effects. Our data show that the task-sharing component of ComHIP is acceptable to patients and healthcare providers, but not to policymakers, and this has implications on the implementation of ComHIP and the design of related task-shifting strategies in Ghana. Although there is no shortage of evidence on the clear need for such task-sharing strategies in LMICs, these challenges preclude their implementation. We recommend meaningful engagement of policy-level gatekeepers, medical and other relevant professional associations to buy into the concept of task-sharing.

## Supplementary information

**Additional file 1:** Study tools. Comprises the study’s five tools used in data gathering.

## Data Availability

The dataset generated and/or analysed during the current study is available in the Harvard Dataverse repository, 10.7910/DVN/JJHOTB.
