# Supplementary material for: Health system challenges to hypertension and related non-communicable diseases prevention and treatment: perspectives from Ghanaian stakeholders
Source: BMC Health Serv Res. 2019 Oct 15;19:693. doi: 10.1186/s12913-019-4571-6 (PMC6792211; doi:10.1186/s12913-019-4571-6)
Supplement: Supplementary file 1 — Additional file 1: Study tools. Comprises the study’s five tools used in data gathering. [file 12913_2019_4571_MOESM1_ESM.docx]

1. **Semi-structured interview guide for health professionals**

**Note to Interviewer-Please remember this is a guide; so please conduct these interviews following the topic guide but allow for flexibility**

Interviews with health care professionals should focus on their expertise and the role they play in the prevention and treatment of HT and other CVDs, as well as understanding their relationship with the patient, how the care is coordinated; the information they provide to the patient; and what are the areas that are needed for improvement. In addition, the interviewer should try to find out what is the health professional’s knowledge of the existent regional, national programmes to prevent and treat HT. The interviewer should also ask questions on the use and implementation of clinical guidelines. The question will be tailored to whether the interviewee is a community worker, nurse, GP or secondary care specialist.

**Table 2 Interview Guide with health care professionals using the example of Hypertension**

|  | **HEALTHCARE PROFESSIONALS’ EXPERIENCE AND TASKS** |  |
| --- | --- | --- |
|  | Can you tell me about your role as a clinician/nurse/specialist? | HCT related |
|  | Can you tell me how your service is organized? | HCT related  Health system related |
|  | Can you describe the other health care professionals you work with? | HCT related |
|  | How is HT diagnosed? What are the key steps? | Condition related |
|  | What treatment do HT patients take? | Therapy related |
|  | Once a patient has been diagnosed what is the prevention information that is provided? | HCT related |
|  | How is prevention organised at primary health care level or the hospital level and how this is coordinated with other organizations? Health Promotion Agency? | Health system related |
|  | **TREATMENT** |  |
|  | Could you please explain to me the steps that a patient follows when they come to you and you suspect HT or another CVDs (Could you please provide two examples, a patient with no complications and a patient with several comorbidities.) | Patient related |
|  | How is this care coordinated with the rest of the team you work with, and with other services (secondary care, primary care)? | HCT related |
|  | What are the things that you think work well? | HCT related |
|  | How do you think you could coordinate care better? What are the biggest challenges? | HCT related |
|  | **CLINICAL GUIDELINES** |  |
|  | What Clinical Guidelines on hypertension exist in Ghana? How are these developed? | Health System related |
|  | What are your views on Clinical Guidelines? Do you think these are useful? | Health System related |
|  | What are some of the barriers/facilitators of implementing clinical guidelines? | Health System related |
|  | **RELATIONSHIPS WITH PATIENTS** |  |
|  | Can you tell me about your interactions with patients? What are challenges to effective communication? | Patient related |
|  | What do you think are the key challenges to adherence to treatment in your own experience? | ALL |
|  | What are the areas where you think it could be done more to inform the patient? How could this be done? | ALL |
|  | **HEALTH SYSTEM ISSUES** |  |
|  | What areas do you think work well in your system regarding NCDs? | Health System related |
|  | Do you think all patients have the same access to care in Ghana? | Health System related |
|  | What areas could be improved to make your work easier and more enjoyable? (Probe for: Is funding a key concern for you? Lack of physical resources? Or the lack of human resources, or the information systems, or poor facilities, or access to care?) | HCT related  Health System related |
|  | What could be done to improve the prevention and treatment for patients? | ALL |
|  | **QUESTIONS ABOUT INTERVENTION** |  |
|  | What is your role within the ComHIP programme? | HCT |
|  | Can you tell me a bit about the level of training you received as part of ComHIP? Do you think it was sufficient? [PROMPT for length and frequency of training] |  |
|  | Can you talk a bit about your experience in the ComHIP programme? | Programme |
|  | What do you think are the biggest strengths of ComHIP? | Programme |
|  | What have you found to be the greatest challenges in the implementation of the programme? | Programme |
|  | How is the programme different than what existed for Hypertension control in Ghana before? Do you think it is better? How? | Programme/HCT |
|  | How have you found the use of tablets while conducting the programme? Have you had any challenges? |  |
|  | Do you think that the programme is successful in increasing awareness of hypertension in Ghana? Do you think it is successful in increasing control of hypertension? Why or why not? | Programme/HCT |
|  | Do you think that the programme is appropriate in this district? | Programme |
|  | Can you give me an example of an instance where one of the participants was unhappy with their participation in the programme? | Programme/Patient |
|  | In your opinion has this programme had an impact on other existing programmes? | Programme/HCT |
|  | **Closing Questions** |  |
|  | Before we wrap up, do you have any final thoughts that you’d like to share? |  |
|  | This brings us to the end of our interview for today. Do you have any final questions? |  |

Challenges and risks

Explore further

Sustainability matters

Explore further

Lessons and best practices

Explore further

1. **Semi-structured Interview guide for policy makers**

**Interview Guide with Policy makers**

**Note to Interviewer-Please remember this is a guide; so please conduct these interviews following the topic guide but allow for flexibility**

This interview needs to be tailored to the expertise of the interviewee. For example if the interviewee is an expert on how the health care system is financed, the questions should focus on this expertise. It is key to identify at the beginning of the interview what areas of expertise the interviewee has if a profile of the interview has not been made available. It is better that each interview is tailored beforehand. Some of the questions that interviews need to cover are described below. The aim is that we should have data on all of these aspects, but being aware that not all interviewees will have the expertise to answer all the questions. We have introduced a suggestion of which expert should be asked what questions. There is some repetition but the purpose is to provide a wide range of questions that can be adapted to the expertise of the interviewee.

The overall information that we need is to understand how ComHIP fits into the existing health system including financing, human resources, IT and monitoring, service delivery; what programmes, strategies exist on NCDs; what are the priority areas and key risk factors; what are the key challenges in implementing such programmes; what are the health system barrier and facilitators; who are the key actors; what are some of the contextual factors; and what are the areas that need improving.

**Table 3 Interview Guide with policy makers**

| **Introductory questions**   - To start, tell me a little about yourself? - Can you tell me about your employment experience in the past 10 years (Based on this we will be able to tailor the questions). - Describe your current profession. - What are your key responsibilities? - What is your experience regarding the prevention and treatment of Hypertension? and other CVDs?   **Organization of the system (broader questions)**   - Is there a national/regional strategy or policy for CVD or HT? - Is there a national registry for NCDs? - Who are the key stakeholders in the prevention and treatment of Hypertension and other CVDs? - How is prevention organised at physician level, hospital level and how this is coordinated with other organizations? (e.g the role of the Health Promotion Agency) - How is the treatment of HT care organized in Ghana? - What are some of the challenges faced in the provision of prevention or treatment for Hypertension? - To what extent is Hypertension a priority? And how has this changed over time?   **Health Systems Issues (ask an expert on the health system about this)**   - What are some of countrywide key health systems reforms that are worth mentioning? - How is the health systems financed in Ghana and specifically, how are NCDs financed? How are pharmaceuticals financed in Ghana? - How is the human resources organized in Ghana? - What is the role of the community in providing prevention and care? - What do you think is the role of the patient on the decision making process? - How do you think you could coordinate prevention and care better? What are the biggest challenges? - What are the key health system barriers that make it difficult to coordinate the prevention and treatment of HT? (Probe for: Is funding a key concern for you? Or the lack of human resources, or the information systems, or poor facilities, or access to care?) - What could be done to improve the prevention and treatment from the patients’ perspective? - Do you think all patients have the same access to care in your country?   **Identifying the contextual factors that influence the design and implementation of NCD programmes (ask a policy expert and health system’s expert)**   - In your opinion, what are the key factors that shape Ghana’s health system today? - What are Ghanas’s *public health* challenges? - What are Ghana’s current *healthcare* challenges? - How do you think these contextual factors influence the implementation of NCD programmes? - Tell us what you know about the policies that have been introduced to facilitate the implementation of NCD programmes? - What do you think are some factors that enable the implementation of NCD programmes in Ghana? - And what do you think are some factors that make implementation challenging? - Are there additional things that you think could help improve the introduction and implementation of NCD programmes? - Prompt: For example, more visible leadership at government level, better education of the health workforce to ensure buy-in, better public education about how prevention of NCDs benefits them, greater input from community partners, greater input from academic partners (via health services/systems research), objective external evaluations   **Exploring in more detail the perspectives of the actors involved in introducing and implementing ComHIP (ask an expert on the health system and NCDs about this)**   - Who do you think are the important parties involved in introducing and implementing ComHIP? - Prompts: Some of these parties include healthcare providers, public authorities, and patients. - What is each party’s role in introducing and implementing ComHIP? - How do each of these actors work together to implement ComHIP? - Do you think that the programme is useful within the context of the Lower Manya Krobo District? - Do you think the programme is acceptable? - Do you think that the programme is working to enhance people’s awareness of hypertension? Why or why not? - Do you think that the programme is working to help people achieve greater hypertension control? Why or why not? - What do you think are the strengths of ComHIP? - What do you think are the weaknesses of ComHIP? - What are some of the barriers to ComHIPs success? - Has ComHIP had an impact on other existing programmes?  Challenges and risksSustainability mattersLessons and best practices **Closing questions**   - What are your hopes for the preventing and managing NCDs as it evolves? - Before we wrap up, do you have any final thoughts that you’d like to share? - This brings us to the end of our interview for today. Do you have any parting questions? |
| --- |

**3. Semi-structured Interview Guide for Hypertensive Patients**

**Note to Interviewer-Please remember this is a guide; so please conduct these interviews following the topic guide but allow for flexibility**

**Administer after obtaining consent:**

|  | **INTRO AND SOCIAL DETERMINANTS OF HEALTH** | **FACTORS** |
| --- | --- | --- |
| 1 | Tell me a bit about yourself. What is your name and what did/do you do for a living? | Social/Economic related |
| 2 | (If retired) When did you stop working? | Social/Economic related |
| 3 | Tell me about your educational background? | Social/Economic related |
| 4 | Do you have a family? Can you tell me about them? | Social/Economic related |
| 5 | How often do you see your family? | Social/Economic related |
| 6 | Tell me about the place you work. What are your job responsibilities? | Social/Economic related |
| 7 | Tell me about the place where you live. What are your living arrangements? | Social/Economic related |
| 8 | Do you have someone who supports you at home? Can you talk a bit about who you live with? | Social/Economic related |
| 9 | Do you have a valid health insurance card? | Health System |
|  | **KNOWLEDGE AND DIAGNOSIS** | **FACTORS** |
| 10 | Can you tell me about your health problems?  (prompt: the one that worries the patient most, then follow up with other, and then ask about HT) | Condition related  Patient related |
| 11 | When were you diagnosed? What led you to seek care? How was hypertension explained to you. | Condition related  Patient related |
| 12 | Why did you decide to seek care? Who helped you in the process? | Condition related  Patient related  Health system related |
| 13 | Can we talk about your experience of this process of seeking care? | Patient related |
| 14 | Where are you seeking care? How did you access services there? | Health system related |
| 15 | To what extent do you think HT is an important disease? | Condition related  Patient related |
| 16 | How much did you know about HT before your diagnosis? What were your information sources, at the time and now? | Patient related |
| 17 | To what extent do you think treatment of hypertension is important? | Patient related  Therapy related |
|  | **PREVENTION, TREATMENT AND MANAGEMENT PRE INTERVENTION**  **Read: Please answer these questions thinking about your situation before enrolling in this programme** | **FACTORS** |
| 18 | Prior to enrolling in the programme, did you seek medical care for hypertension (if answer is no, go to Q33) |  |
| 19 | Prior to enrolling in this programme did you receive any advice on preventive measures? From whom did you receive such advice? | Health system related |
| 20 | Prior to this programme, did you change your behaviour (diet, exercise) since knowing about the condition? [Probe for barriers/facilitators to change] | Patient related |
| 21 | Do you remember when you first started treatment? Can you tell me about your experience? | Therapy related |
| 22 | Did you have to pay anything out-of-pocket for the treatment? | Therapy related |
| 23 | Did you have to pay anything to travel to the health care facility? | Social/Economic related |
| 24 | Did you face difficulties during the process?  (Prompt: reception, waiting times) | Health system related  Patient related |
| 25 | If you had a complication did you know what to do? | Health system related |
|  | **INTERVENTION.**  **Read out: When answering these questions think about your experiences in the Com-HIP programme** |  |
| 26 | What advice have you received on lifestyle measures to control your hypertension Do you feel it was reasonable? | Programme |
| 27 | Have you changed your behaviour (diet, exercise) since knowing about the condition? [Probe for barriers/facilitators to change] | Programme |
| 28 | Can you tell me about your experience in the Programme? [Prompt: Availability of resource/drugs, attitudes of health care professionals, ease of accessing care, waiting times, transport problems] | Programme |
| 29 | Does anyone (e.g. a family member or organization) help you with taking the treatment? | Programme |
| 30 | Do you feel the treatment has an effect? DO you know if the treatment has been changed since you have been in the programme [Probe for side effects – what they do if they experience side effects] | Programme |
| 31 | How would you describe your relationship with your health care providers? Do you have any examples? [PROMPT: attentiveness, do they listen, asked if you had any concerns] | Programme |
| 32 | How far did you have to travel to receive care? Did you have to pay to travel for care? Would the distance or cost keep you from accessing services again? | Programme |
| 33 | How were the health care facilities your visited? [PROMPT for cleanliness, well stocked, have resources, attentive staff, waiting times] | Programme |
| 34 | Did you face difficulties during the process? | Programme |
| 35 | Do you know what to do if you have any clinical complications? What potential complications did your provider discuss with you? | Programme |
| 36 | Were you asked to talk about any problems with your medicines or their effects? Do you feel they heard your concerns? | Programme |
| 37 | What in the process of treatment could have been handled better? | Programme |
| 38 | Do you find the text messages useful? Appropriate? Was there anything you found difficult to understand? | Programme |
| 39 | Where do you get your regular supply of medication? Have you had problems receiving the medication you need? Were you able to fill your last prescription. If not, why not [PROMPT: availability of drugs, cost? | Programme |
| 40 | Do you have a particular health professional who is looking after you and who knows you well? | Programme |
| 41 | How would you assess your communication with the nurses and other health care professionals you have encountered? | Programme |
| 42 | How would you assess your experiences with the licensed chemical sellers? | Programme |
| 43 | Do you feel your health care providers respect your opinions? | Programme |
| 44 | To what extent do you feel you are kept informed about your treatment? | Programme |
| 45 | In the last two weeks, how many times have your forgotten to take your medication? Why did you forget? What did you do when you realized you had forgotten? | Programme |
| 46 | Is there anyone or any help you could have received that would have helped you to remember taking your medication? | Programme |
| 47 | Do you use alternative, local, or faith based medicines? Which is these medications (traditional or medical) do you believe is more effective for the treatment of hypertension? | Programme |
| 48 | Do you have someone else who provides you with a regular supply of medications? What are these medications for? Do you tell the health care professionals about them? |  |
| 49 | Comparing your experiences in ComHIP with what you experienced before, what are they main differences? [Prompt: availability of medications, Health care professionals, access to care, communication, cost] |  |
|  | **Closing Questions** |  |
| 50 | Before we wrap up, do you have any final thoughts that you’d like to share? |  |
| 51 | This brings us to the end of our interview for today. Do you have any final questions? |  |

1. **Focus Group Discussion Guide – Hypertensive Patients**

**Note to Interviewer-Please remember this is a guide; so please conduct the FGD following the topic guide but allow for flexibility**

**Introduction**

Greetings and thank you for coming.

We are researchers from the University of Ghana and London School of Hygiene & Tropical Medicine. We are part of the ComHIP project that you are enrolled in for hypertension care. We are going to give you information and invite you to be part of a focus groups to help us understand your experiences in this programme. If there is anything here, that you do not understand, please ask me to stop as we go through the information and I will take time to explain. If you have questions later, you can ask them of me or of another researcher.

You are part of the ComHIP programme which is aimed at lowering your blood pressure. We would like to hear from you about your experiences in the programme, and tell us if you think this programme is useful, and how it has affected you. What we find out here can help us to make the programme more acceptable and help us to find any issues with the programme.

We are asking you today to take part in a focus group. That is a group discussion that will be led by a member of our team, and 5-6 other people who are participating in the programme.

Your participation in this focus group is completely voluntary. It is your choice whether to participate or not. If you choose not to participate all the services you receive at this Centre will continue and nothing will change.

The focus group will be led by a member of our team, and will last approximately one and a half hours. The leader will ask you some questions about the care that you received, and if you felt that it was appropriate. If you feel uncomfortable at any time, you can leave. If you have any questions at any time, you can ask the leader to explain things. No one else but the people who take part in the discussion and leaders will be present during this discussion. The discussion will be recorded, but no-one will be identified by name on the tape. The information recorded is confidential, and no one outside of our team will have access to the tapes. The tapes will be destroyed after the data are analysed.

We will ask you and others in the group not to talk to people outside the group about what was said in the group. We will, in other words, ask each of you to keep what was said in the group confidential. You should know, however, that we cannot stop or prevent participants who were in the group from sharing things that should be confidential

We will not share any data about you with anyone outside of the research team, and any quotes that we use will be anonymised, and we will not use information about your age, gender or anything else that can identify you. If at any point you decide that do not want the information that you provided used by our team, we will not use it.

Is there anything here that you do not understand?

- We would like you to sign an informed consent form to affirm your voluntary participation in this discussion.
- [*Pass out informed consent form to participants*].

**Background data for each participant**

Date:

Time:

Location:

Moderator:

**Participants’ Demographic Information**

| **Initials of participants** | **Age** | **Sex:**  **M/F** | **Ethnicity** | **Native language** | **Add during training –as appropriate** | **Add during training –as appropriate** | **Add during training –as appropriate** |
| --- | --- | --- | --- | --- | --- | --- | --- |
| 1. |  |  |  |  |  |  |  |
| 2. |  |  |  |  |  |  |  |
| 3. |  |  |  |  |  |  |  |
| 4. |  |  |  |  |  |  |  |
| 5. |  |  |  |  |  |  |  |
| 6. |  |  |  |  |  |  |  |
| 7. |  |  |  |  |  |  |  |
| 8. |  |  |  |  |  |  |  |
| 9. |  |  |  |  |  |  |  |
| 10. |  |  |  |  |  |  |  |

Ground rules

- Before we begin, we would like you to know that there are no prejudiced judgments against anyone here. Feel free to express your ideas, opinions, or experiences.
- As you may have noticed, there is a tape recorder placed on the table. We hope that having a tape recorder will not make you feel uncomfortable. This is to ensure we capture all your ideas and that we do not miss any of the valuable information the group may offer. We also have a note taker who will be writing your response to make sure that we capture what you are saying correctly.
- Before we begin, does anyone have any questions or comments?

FGD Guide

- Do you feel that hypertension is an important health issue? Why or why not?
- Is it important to take your medications every day? Has your opinion on this changed since being involved in the programme?
- Can you talk about your experiences in the programme?
  - What have you learned by being involved with the ComHIP programme?
  - How does the comHIP programme compare to your experiences with previous experiences with other health services?
  - Do you feel that the health care professionals that you have seen listen to you and your concerns?
  - Were the challenges when you first enrolled different to those after you had been enrolled for a while?
- Have you experienced any unexpected consequences since starting ComHIP?
  - If you have a problem with any of your medications, do you know who to talk to?
- Does this programme empower you to take control of the management of your hypertension?—control of your own health?
- What is the best part about being involved in the programme?
- What is the worst part about being involved in the programme?
- Would you recommend this programme to other colleagues? [Probe why and how for yes, or no responses)
- **Recommendation for improving ComHIP/hypertension prevention and treatment services**
- How can we improve upon ComHIP or hypertension prevention and treatment services
- What suggestions do you have for improving these services?
- What areas could be improved?

**Thank you for your time and contribution to this discussion**

(Moderator: Encourage off-the-record comments).

1. **Focus Group Discussion Guide – License Chemical Sellers (LCS)**

**Note to Interviewer-Please remember this is a guide; so please conduct the FGD following the topic guide but allow for flexibility**

**Introduction**

Greetings and thank you for coming.

We are researchers from the University of Ghana and London School of Hygiene & Tropical Medicine. We are part of the ComHIP project that you are enrolled in for hypertension care. We are going to give you information and invite you to be part of a focus groups to help us understand your experiences in this programme. If there is anything here, that you do not understand, please ask me to stop as we go through the information and I will take time to explain. If you have questions later, you can ask them of me or of another researcher.

You are part of the ComHIP programme which is aimed at lowering your blood pressure. We would like to hear from you about your experiences in the programme, and tell us if you think this programme is useful, and how it has affected you. What we find out here can help us to make the programme more acceptable and help us to find any issues with the programme.

We are asking you today to take part in a focus group. That is a group discussion that will be led by a member of our team, and 5-6 other people who are participating in the programme.

Your participation in this focus group is completely voluntary. It is your choice whether to participate or not. If you choose not to participate all the services you receive at this Centre will continue and nothing will change.

The focus group will be led by a member of our team, and will last approximately one and a half hours. The leader will ask you some questions about the care that you received, and if you felt that it was appropriate. If you feel uncomfortable at any time, you can leave. If you have any questions at any time, you can ask the leader to explain things. No one else but the people who take part in the discussion and leaders will be present during this discussion. The discussion will be recorded, but no-one will be identified by name on the tape. The information recorded is confidential, and no one outside of our team will have access to the tapes. The tapes will be destroyed after the data are analysed.

We will ask you and others in the group not to talk to people outside the group about what was said in the group. We will, in other words, ask each of you to keep what was said in the group confidential. You should know, however, that we cannot stop or prevent participants who were in the group from sharing things that should be confidential

We will not share any data about you with anyone outside of the research team, and any quotes that we use will be anonymised, and we will not use information about your age, gender or anything else that can identify you. If at any point you decide that do not want the information that you provided used by our team, we will not use it.

Is there anything here that you do not understand?

- We would like you to sign an informed consent form to affirm your voluntary participation in this discussion.
- [*Pass out informed consent form to participants*].

**Background data for each participant**

Date:

Time:

Location:

Moderator:

**Participants’ Demographic Information**

| **Initials of participants** | **Age** | **Sex:**  **M/F** | **Ethnicity** | **Native language** | **Add during training –as appropriate** | **Add during training –as appropriate** | **Add during training –as appropriate** |
| --- | --- | --- | --- | --- | --- | --- | --- |
| 1. |  |  |  |  |  |  |  |
| 2. |  |  |  |  |  |  |  |
| 3. |  |  |  |  |  |  |  |
| 4. |  |  |  |  |  |  |  |
| 5. |  |  |  |  |  |  |  |
| 6. |  |  |  |  |  |  |  |
| 7. |  |  |  |  |  |  |  |
| 8. |  |  |  |  |  |  |  |
| 9. |  |  |  |  |  |  |  |
| 10. |  |  |  |  |  |  |  |

Ground rules

- Before we begin, we would like you to know that there are no prejudiced judgments against anyone here. Feel free to express your ideas, opinions, or experiences.
- As you may have noticed, there is a tape recorder placed on the table. We hope that having a tape recorder will not make you feel uncomfortable. This is to ensure we capture all your ideas and that we do not miss any of the valuable information the group may offer. We also have a note taker who will be writing your response to make sure that we capture what you are saying correctly.
- Before we begin, does anyone have any questions or comments?

Begin:

One of the assumption in ComHIP was that the LCS would make money indirectly by being involved in ComHIP through greater visibility within the community and people making purchases when attending LCS for screening. Followup or prescription refills. The purpose of the focus groups is to see if this is true.

Questions for discussion:

- Have you been trained by ComHIP
- In your absence who provides service?
- What happens if there is no one trained in the LCS, and someone comes in for screening?
- What has been your experience with comHIP
- How has comHIP changed the way people use your services?
- Have you found that more people in the community are using any of your services (related and not related to the programme)
- Would you be interested in continuing to be involved in screening, monitoring blood pressure, and provide advice of people in the community? Why or why not?
- Have your revenues increased by being involved in comHIP?
- What further training would you like to have in order to help BP control in the community?
- What is the biggest challenge you have had since being involved in the programme?
- What is the best part of being involved in the programme?
- Do you think you should be allowed to stock and prescribe medications for controlling hypertension? Why or why not?
- Would you recommend this programme to other colleagues? [Probe why and how for yes, or no responses)
- **Recommendation for improving ComHIP/hypertension prevention and treatment services**
- How can we improve upon ComHIP or hypertension prevention and treatment services
- What suggestions do you have for improving these services?
- What areas could be improved?

**Thank you for your time and contribution to this discussion**

(Moderator: Encourage off-the-record comments).
